# Long-wave opsin involved in body color plastic development in *Nilaparvata lugens*

**DOI:** 10.1186/s12864-023-09470-7

**Published:** 2023-06-26

**Authors:** Jia-Bao Lu, Ze-Dong Li, Zhuang-Xin Ye, Hai-Jian Huang, Jian-Ping Chen, Jun-Min Li, Chuan-Xi Zhang

**Affiliations:** 1 State Key Laboratory for Managing Biotic and Chemical Threats to the Quality and Safety of Agro-products, Key Laboratory of Biotechnology in Plant Protection of MARA and Zhejiang Province, Institute of Plant Virology, Ningbo University, Ningbo, 315211 China; 2 Institute of Insect Science, Zhejiang University, Hangzhou, 310058 China

**Keywords:** Opsin, Melanization, Body color, RNAi, Transcriptome, Genome, *Nilaparvata lugens*

## Abstract

**Background:**

As one of the components of visual photopigments in photoreceptor cells, opsin exhibits different spectral peaks and plays crucial roles in visual function. Besides, it is discovered to evolve other functions despite color vision. However, research on its unconventional function is limited nowadays. With the increase in genome database numbers, various numbers and types of opsins have been identified in insects due to gene duplications or losses. The *Nilaparvata lugens* (Hemiptera) is a rice pest known for its long-distance migration capability. In this study, opsins were identified in *N. lugens* and characterized by genome and transcriptome analyses. Meanwhile, RNA interference (RNAi) was carried out to investigate the functions of opsins, and then the Illumina Novaseq 6000 platform-based transcriptome sequencing was performed to reveal gene expression patterns.

**Results:**

Four opsins belonging to G protein-coupled receptors were identified in the *N. lugens* genome, including one *long-sensitive opsin* (*Nllw*) together with two *ultraviolet-sensitive opsins* (*NlUV1/2*) and an additional new *opsin* with hypothesized UV peak sensitivity (*NlUV3-like*). A tandem array of *NlUV1/2* on the chromosome suggested the presence of a gene duplication event, with similar exons distribution. Moreover, as revealed by spatiotemporal expression, the four *opsins* were highly expressed in eyes with age-different expression levels. Besides, RNAi targeting each of the four opsins did not significantly affect the survival of *N. lugens* in phytotron, but the silencing of *Nllw* resulted in the melanization of body color. Further transcriptome analysis revealed that silencing of *Nllw* resulted in up-regulation of a *tyrosine hydroxylase* gene (*NlTH*) and down-regulation of an *arylalkylamine-N-acetyltransferases* gene (*NlaaNAT*) in *N. lugens*, demonstrating that *Nllw* is involved in body color plastic development via the tyrosine-mediated melanism pathway.

**Conclusions:**

This study provides the first evidence in a Hemipteran insect that an opsin (*Nllw*) takes part in the regulation of cuticle melanization, confirming a cross-talk between the gene pathways underlying the visual system and the morphological differentiation in insects.

**Supplementary Information:**

The online version contains supplementary material available at 10.1186/s12864-023-09470-7.

## Background

The visual system is essential for insects to collect abundant information from their surroundings and respond appropriately, including making choices among alternative behaviors, foraging, and mating [1–4]. Generally, the spectral sensitivity of photoreceptor cells is determined by the photopigment, a complex of chromophore and opsin [5]. The chromophore, usually the 11-*cis*-retinal derived from vitamin A, forms a covalent bond with a lysine residue (K) in the opsin, a light-activated G-protein coupled receptor, via a Schiff base [6–8]. Opsins can be usually categorized into three groups based on their spectral peaks, namely, long-wavelength-sensitive (LW), blue-sensitive (B), and ultraviolet (UV)-sensitive proteins [9]. The majority of insects possess this ancestral trichromatic visual system, such as *Drosophila melanogaster* (Diptera) [10, 11], *Apis mellifera* (Hymenoptera) [12], *Spodoptera exigua* (Lepidoptera) [13], and *Nephotettix cincticeps* (Hemiptera) [14]. As more and more genome databases have been constructed, insects are found to possess various numbers or types of opsins due to gene duplications or losses [15]. For instance, one UV-sensitive, five B-sensitive, and up to ten LW-sensitive opsins are present in *Sympetrum frequens* (Odonata) [16]; meanwhile, the ancestral B-sensitive opsin is lost in Coleoptera insects, but the trichromacy at least twelve times is regained by duplicating the UV-sensitive and LW-sensitive opsin genes [17, 18].

In *Drosophila*, Rh1-6 opsins are expressed in the compound eyes, among which, Rh1 long-sensitive opsin is expressed in photoreceptor cells 1–6, Rh3/4 UV-sensitive opsins in photoreceptor 7 cells, whereas photoreceptor 8 cells express the Rh5 opsin for blue light detection and the Rh6 opsin for green light detection [11, 19, 20]. Another opsin, Rh7, found in the central brain, helps regulate circadian light entrainment by circadian pacemaker neurons [21]. In addition to the above ancestral visual functions, insect opsins have also evolved additional functions that are crucial for insect survival and fitness [22, 23]. For instance, in *Drosophila*, Rh1 plays a crucial role in the initiation of thermosensory signaling cascades for thermotaxis [24]; additionally, two other opsins, Rh5 and Rh6, function in the brain and body wall to support thermal preference during the late-3rd instar larvae [25]; furthermore, Rh6 is also responsible for the cooling-induced activation of bitter gustatory receptor neurons [26]; and opsins are also suggested to be linked to morphological differentiation, like the evolution of distinct yellow wing pigment along with a second UV opsin in *Heliconius* butterflies [27]. Despite the extensive research regarding the impact of opsins on the visual function across various insect species [23], the unconventional roles of these opsins in insects remain largely mysterious.

The *Nilaparvata lugens* (Hemiptera), belonging to the Delphacidae family, is the most destructive rice pest in Asia. It is particularly notable for its wing dimorphism and the capability of long-distance migration, allowing it to exploit its exclusive rice host in temperate regions and inflict significant damage on rice crops [28]. A previous study has identified three opsin genes in *N. lugens* genome, including one LW- and two UV - absorbing opsins (*Nllw*, and *NlUV1*/*2*, respectively) [14]. However, it is still unknown whether there are other functions except for visual sensing. In this study, opsin genes in the *N. lugens* were systematically investigated based on transcriptomic and genomic data, and an additional new *opsin* with hypothesized UV peak sensitivity (*NlUV3-like*) was identified. Thereafter, the functions of these four opsin genes were investigated, respectively. According to our results, *Nllw* took part in the melanization of body color and might be involved in the tyrosine-mediated cuticle melanism pathway. As far as we know, this study is the first to reveal the unconventional function of opsins in Hemiptera and suggests a cross-talk between gene pathways underlying the visual system and the morphological differentiation in insects.

## Results

### Identification of opsin genes from the genome and transcriptome databases

To identify the potential *opsin* genes in *N. lugens*, the *Drosophila* ninaE protein sequence (NP_524407.1) was used as a query to search the corresponding genome and transcriptome databases of *N. lugens* [29, 30]. As a result, altogether four *opsin* genes were identified, including the previously reported *Nllw* (GenBank accession number: AB761147.1), *NlUV1* (GenBank accession number: AB761148.1), *NlUV2* (GenBank accession number: AB761149.1), and an additional paralog. Blast analysis of the paralog revealed a 99% identity with a UV-sensitive opsin predicted from an *N. lugens* genome sequence (GenBank accession number: NC_052505.1), indicating that a *UV-sensitive-like* *opsin* (*NlUV3-like*) (GenBank accession number: XP_022205432) was detected in *N. lugens*.

### Sequence and phylogenetic analysis

**Fig. 1:**
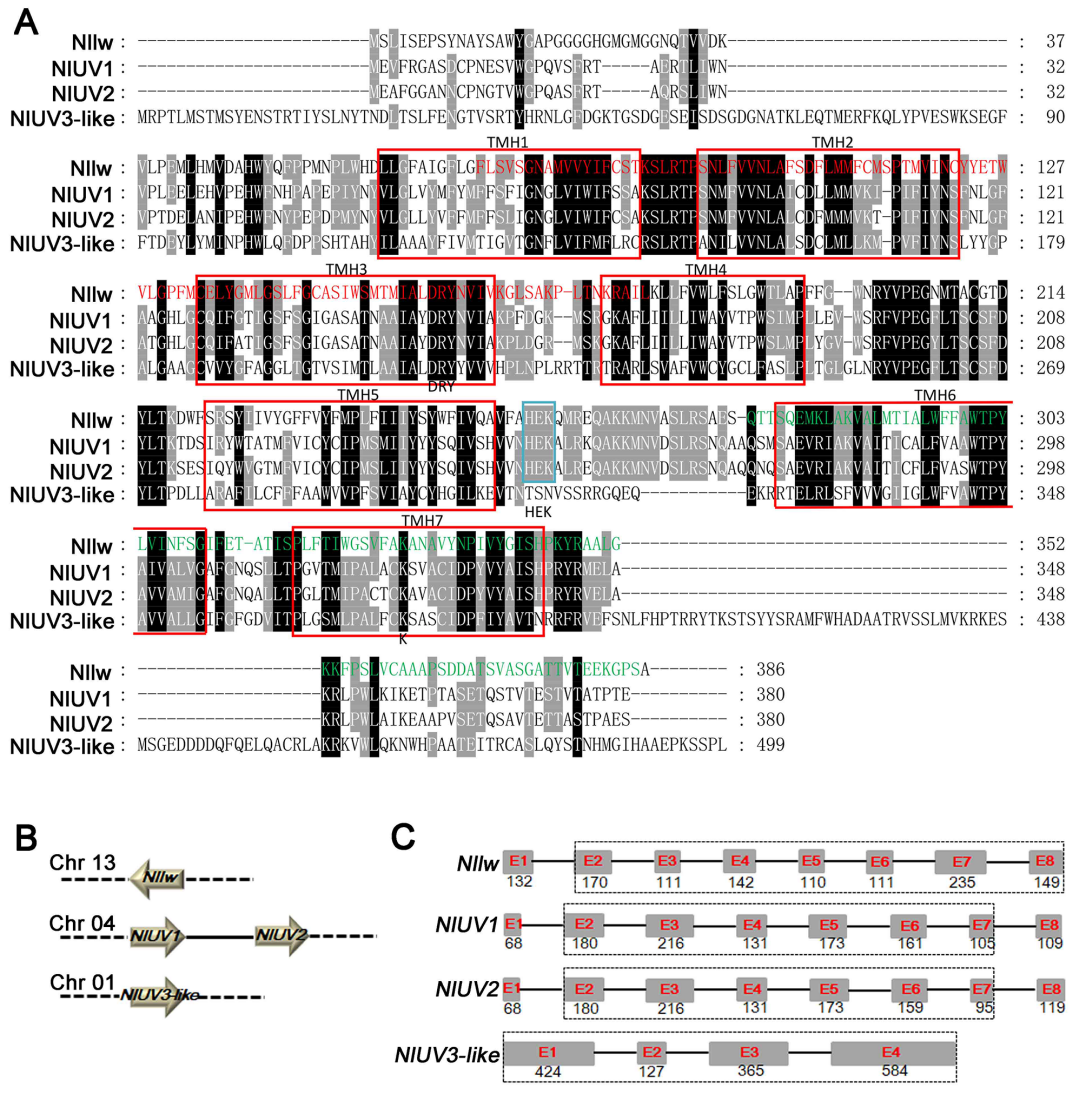
Sequence characteristics analysis. **A**. Alignment of the amino acid sequences of Nllw, NlUV1/2, and NlUV3-like. Seven transmembrane α-helices (TMH1-7) are indicated with red frames; The conserved motif histidine-glutamine-lysine (HEK) is denoted by a blue frame; The conserved lysine (K) in TMH7 is marked below; Black background: 100% conserved amino acid sites; Gray background: 80% conserved amino acid sites. Red and green words: the 1st and 2nd regions for RNAi against *Nllw*, respectively. **B**. An ideogram of *Nllw*, *NlUV1/2*, and *NlUV3-like* on three chromosomes. Chr 13/04/01: chromosome 13/04/01; The arrow representing *Nllw* pointing to the left: means the *Nllw* transcript is in the opposite direction to Chr 13; The arrow representing *NlUV1/2* and *NlUV3-like* pointing to the right means the *NlUV1/2* and *NlUV3-like* transcripts are in the same direction to Chr 04 and 01; Solid line means tandem replicates of *NlUV1*/*2*. **C**. The distribution of *Nllw*, *NlUV1/2*, and *NlUV3-like* coding sequences within the genomic sequences. Gray boxes indicate exons (E), and the width of each box is proportional to the size of the corresponding exon. Figures below each exon are the sizes of corresponding exons. Black lines represent introns, which are not proportional to the size of the corresponding introns. The location of seven transmembrane α-helices on exons is marked with black dotted frames

**Fig. 2:**
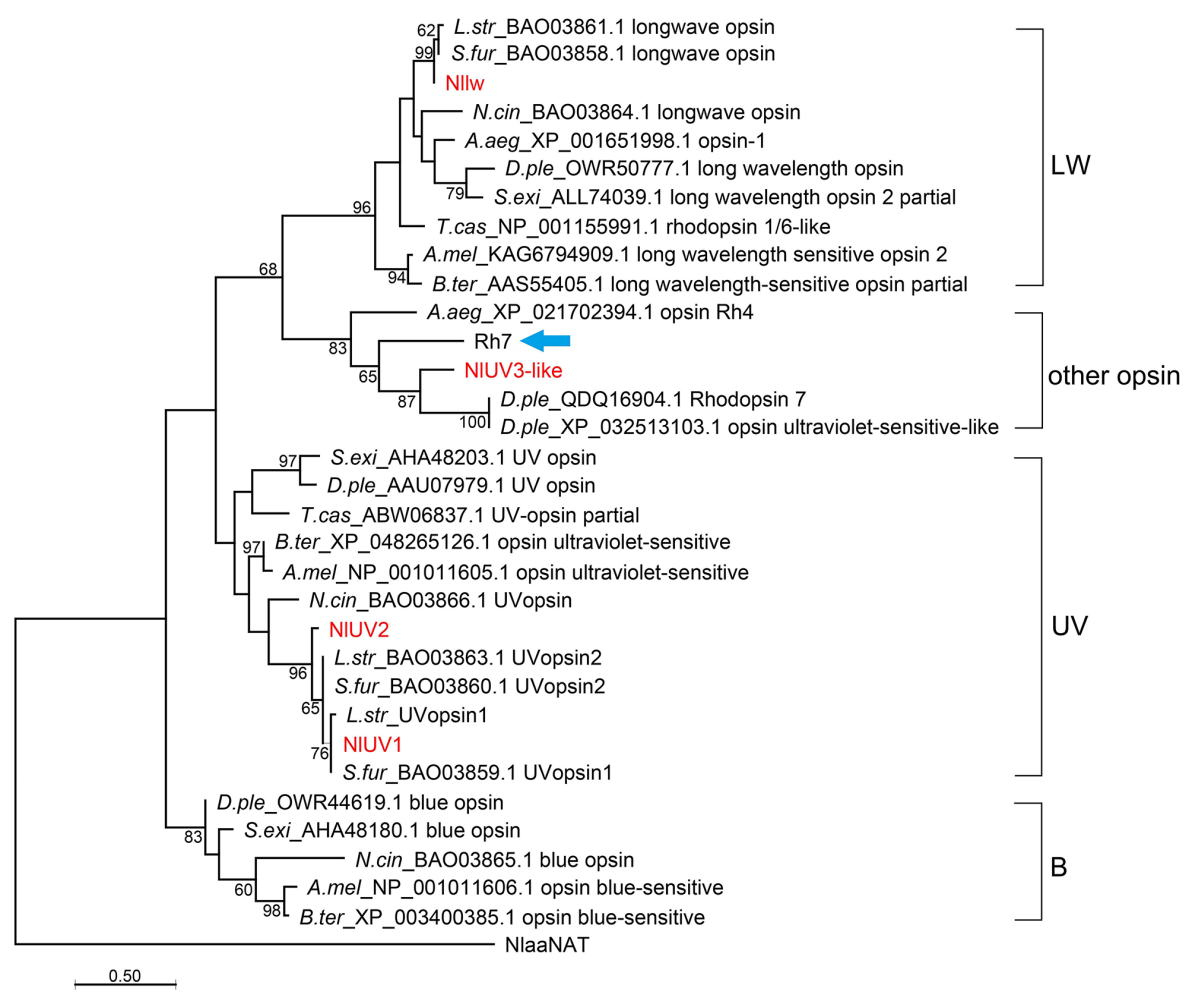
Phylogenetic analysis. A phylogenetic tree with the amino acid sequences of Nllw, NlUV1/2, NlUV3-like, and 28 homologs from other insects. The names represent the abbreviated Latin name – GenBank accession number as well as functional annotation. LW, UV, B: long-wave-, ultraviolet-, and blue-sensitive opsin; Blue arrow: Rh7 in *Drosophila*. Detailed information regarding the 28 homologs is listed in Supplementary Table 3. Only > 50 bootstrap values are shown

The four opsins, Nllw, NlUV1/2, and NlUV3-like were predicted to be G protein-coupled receptors with seven transmembrane α-helices (TMH1-7) [6] and contained a conserved K in TMH7, which covalently binds to the chromophore [7, 8]. A total of 386, 380, 380, and 499 amino acids (aa) peptides were predicted in Nllw, NlUV1/2, and NlUV3-like, respectively. The conserved motif histidine-glutamine-lysine (HEK) involved in G-protein binding [31] was present at the C-terminal side of TMH5 of Nllw and NlUV1/2, but not in NlUV3-like (Fig. 1A). Moreover, these opsins were located on different chromosomes in the *N. lugens* genome, with *Nllw* and *NlUV3-like* on chromosomes (Chr) 13 and 01, separately; whereas *NlUV1*/*2* with tandem distribution on Chr 04 (Fig. 1B). Structural analysis revealed differences in the number of exons spanning TMH1-7, namely, 2nd − 8th (E2-8) in *Nllw*, 2nd − 7th (E2-7) in *NlUV1/2*, and all the four exons (E1-4) in *NlUV3-like* (Fig. 1C). Furthermore, a phylogenetic tree analysis suggested that the Nllw, NlUV1, and NlUV2 clustered well with the long-wave opsins, UVopsin1, and UVopsin2 in *Sogatella furcifera* (Hemiptera) and *Laodelphax striatellus* (Hemiptera), respectively, which was consistent with a previous study [14]. However, the NlUV3-like was distinct from NlUV1/2, which demonstrated strong clustering with the Rh7 (GenBank accession number: NP_524035.2) in *D. melanogaster*, with Rh4 (GenBank accession number: XP_021702394.1) in *Aedes aegypti* (Diptera), as well as with Rhodopsin7 (GenBank accession number: QDQ16904.1) and a UV-like opsin (GenBank accession number: XP_032513103.1) in *Danaus plexippus* (Lepidoptera) (Fig. 2). Overall, the sequence characteristics, Chr location, gene structure, and phylogenetic analysis highlights that Nllw, NlUV1/2, and NlUV3-like are divided into three distinct subfamilies. And the emergence of NlUV1/2 is likely attributed to the duplication of a common ancestor opsin. Meanwhile, NlUV3-like shares homology with other opsin variants, such as Rh7 in *D.melanogaster*, which helps regulate circadian light entrainment in circadian pacemaker neurons [21].

### Spatiotemporal expression patterns

**Fig. 3:**
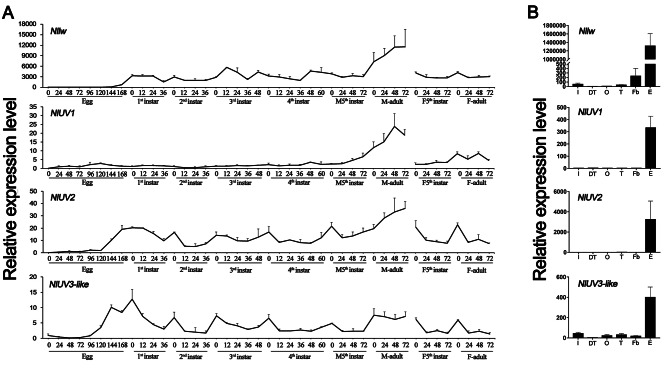
Spatiotemporal expression patterns of *Nllw*, *NlUV1/2*, and *NlUV3-like*. **A**. Age expression patterns of *Nllw*, *NlUV1/2*, and *NlUV3-like* with transcript abundances. **B**. Tissue expression patterns of *Nllw*, *NlUV1/2*, and *NlUV3-like*. I: integument; DT: digestive tract; O: ovary; T: testis; Fb: fat body; E: eye. For **A** and **B**, data represent the means of three biological replicates, (means + SD), and raw data are provided in Supplementary Table 5

Temporal expression patterns were analyzed with the fragments per kilobase per million mapped fragment (FPKM) values based on the transcriptome sequencing data. According to our results, *Nllw* was expressed continuously after the hatching of the 1st instar nymph, with relatively stable expression levels in the nymph and female adult stages, but it reached the highest expression level in the male adult stage. For *NlUV1/2* and *NlUV3-like*, the expression patterns varied obviously. The expression levels of *NlUV1*/*2* peaked in the male adult stage, similar to *Nllw*, but *NlUV1* was barely expressed from the egg stage to the 4th instar nymph stage and showed low expression in the female adult stage. *NlUV2*, on the other hand, exhibited continuous expression from late embryogenesis, a stage when the cuticle of the 1st instar nymph began to form [32]. Besides, *NlUV2* was periodically expressed with molting, with high levels being detected at the beginning and the end of each instar. Whereas *NlUV3-like* showed a similar periodic expression pattern to *NlUV2*, the expression level of *NlUV3-like* peaked in the hatching stage rather than in the male adult period (Fig. 3A). Overall, *Nllw*, *NlUV1/2*, and *NlUV3-like* exhibited exceptionally high expression levels in eyes (Fig. 3B), indicating that all the four opsins are involved in visual processing, albeit with the possibility of divergent functions depending on age-related expression patterns.

### *Nllw* is associated with melanin-type pigment

**Fig. 4:**
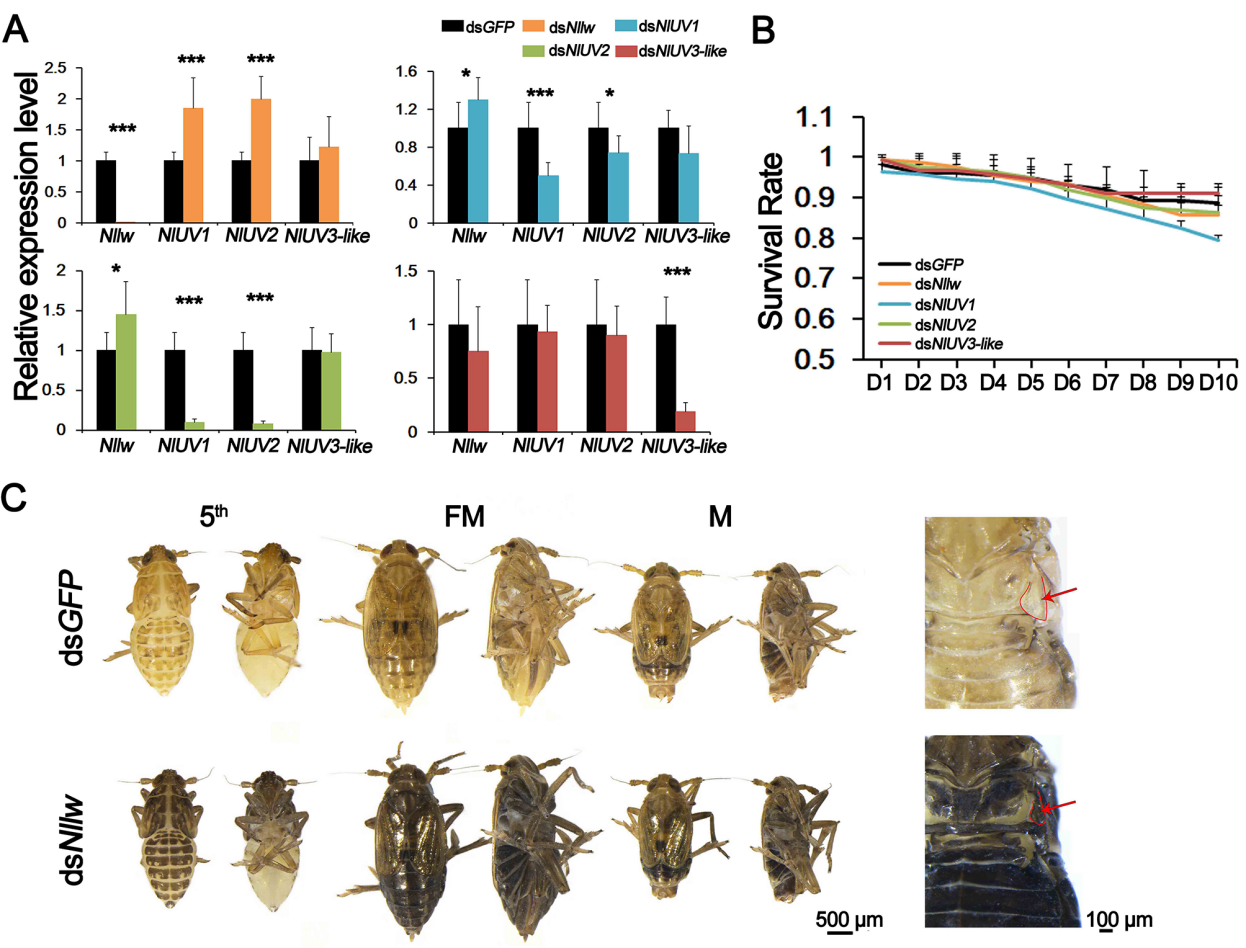
RNAi against each of *Nllw*, *NlUV1/2*, and *NlUV3-like*. **A**. Relative expression levels of *Nllw*, *NlUV1/2*, and *NlUV3-like* after RNAi against each target gene, ds*GFP* was used as the control group. **B**. The survival rates of *N. lugens*. For **A** and **B**, data represent the means of three biological replicates (means + SD). A two-tailed unpaired *t*-test was applied. *P < 0.05 and ***P < 0.001 indicate significant differences compared with the ds*GFP* control group. **C**. Melanism of *N. lugens* after knock-down of *Nllw*. 5th: the 5th instar nymph; FM: female adule; M: male adult. RNAi was performed with the 4th instar nymph. In the ds*Nllw*-treated group, when *N. lugens* developed into the 5th instar, the rigid cuticle of the mesothorax and abdominal dorsal cuticle, leg, wing bud, and head became darker; when *N. lugens* eclosed into adult, melanin-type pigments occurred in almost the whole body surface, except for soft parts of the leg bases of the mesothorax. The short-hind wing which was marked with red line and arrow became darker. In the ds*GFP*-treated control group, *N. lugens* usually remained brown, with a little darker in male adult, and the hind-wing of adult was light color

The expression levels of *Nllw*, *NlUV1/2*, and *NlUV3-like* were detected in all the RNAi-treated groups (Fig. 4A). The target genes were all effectively silenced by RNAi with the early 4th instar nymph *N. lugens*, compared with the ds*GFP* control group. Moreover, the expression levels of the other three opsins were also detected with each RNAi treatment. When *Nllw* was knocked-down, the expression levels of *NlUV1*/*2* were both significantly up-regulated. Conversely, silencing of either *NlUV1*/*2* resulted in a significant up-regulation of *Nllw*, suggesting a possible functional complementation between *Nllw* and *NlUV1*/*2*. However, the down-regulation of either *NlUV1*/*2* led to the silencing of the other one, probably attributed to their 77.97% sequence similarity. It is noteworthy that the silencing of *NlUV3-like* did not impact the expression of *Nllw* and *NlUV1*/*2*. Similarly, the silencing of each of *Nllw* and *NlUV1*/*2* did not affect the expression of *NlUV3-like*, indicating that the expression of *NlUV3-like* is independent of *Nllw* and *NlUV1*/*2*. Among the four opsins in *N. lugens*, RNAi against each target gene did not lead to an apparent lethal phenotype, and there was no significant difference in survival rate compared with the ds*GFP* control group (Fig. 4B). However, an unconventional function was observed in the ds*Nllw*-treated group (Fig. 4C). To be specific, gradual accumulation of melanin occurred when *Nllw* was knocked-down with the 4th instar nymph. When *N. lugens* developed into the 5th instar, the rigid cuticles of the mesothorax, abdominal dorsal cuticle, leg, wing bud, and head became darker; while the other soft and flexible cuticles, including the abdominal ventral plate as well as the segmacoria, were only slightly affected. When *N. lugens* further developed into adults, melanin-type pigments occurred on almost the whole body surface, except for soft parts of leg bases of the mesothorax. In addition, the short-hind wings also became darker. While in ds*GFP*-treated control group, *N. lugens* usually remained brown, with only slight darkening in male adult, but the darkening degree was far less than the ds*Nllw*-treated group *N. lugens*, and the hind-wing of adult was light color in general. To avoid the off-target effect of RNAi, the other non-overlapping RNAi region of ds*Nllw* was designed (Fig 1A). RNAi in two different regions featured similar phenotypes, demonstrating that off-targeting did not occur in this experiment.

### RNA-seq of the transcriptomes revealed potential downstream genes of *Nllw* in the melanism pathway

**Fig. 5:**
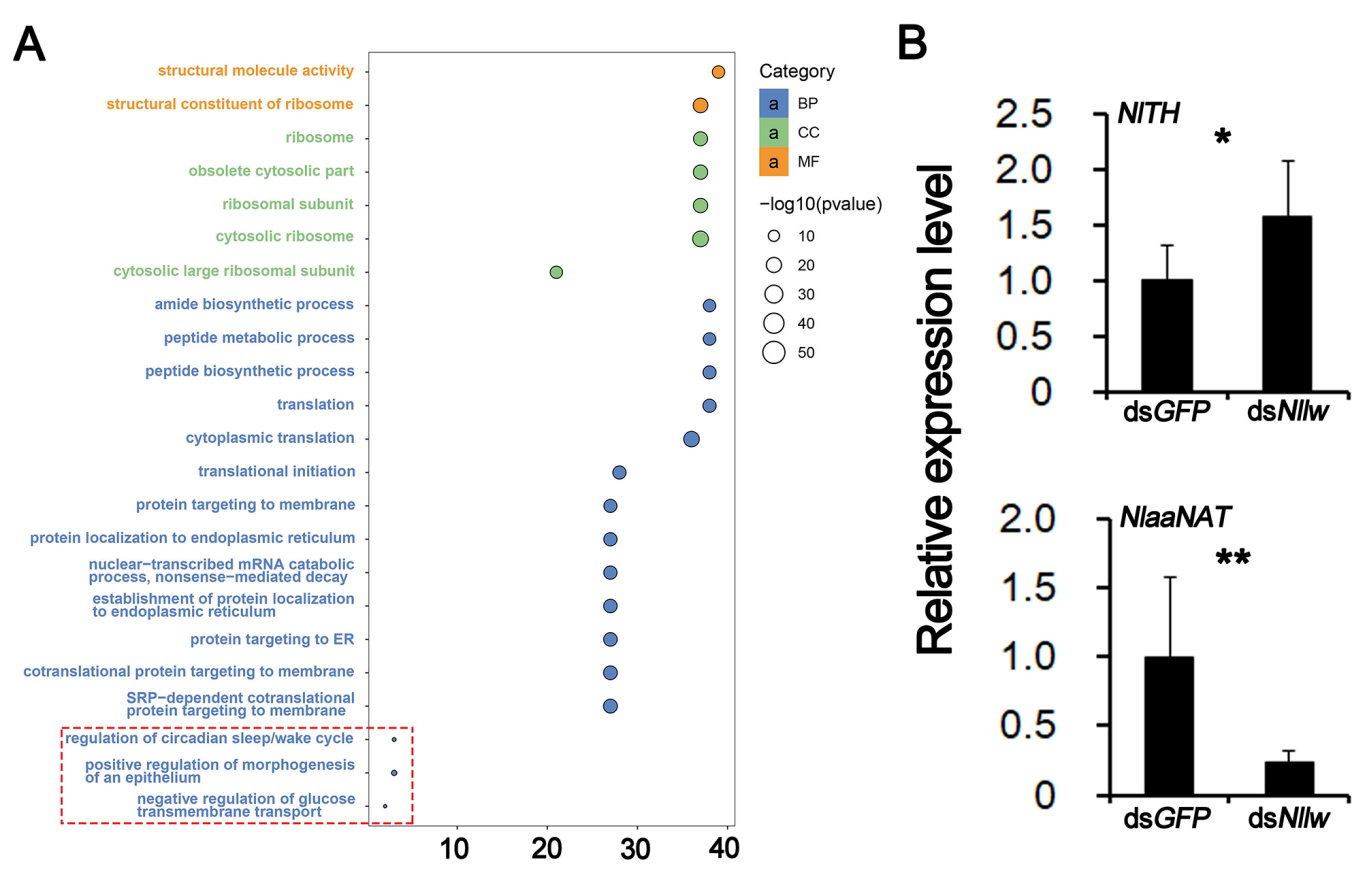
Analysis of potential downstream genes of *Nllw*. **A**. Gene Ontology (GO) analysis after RNAi against *Nllw*. All of the 3 down-regulated GO terms are marked with a red dotted frame; The others are the top 20 up-regulated GO terms. The result is analyzed with three biological replicates. ds*GFP* is used as the control group. **B**. qRT-PCR verification of *NlTH* and *NlaaNAT* after RNAi against *Nllw*. Data represent the means of three biological replicates (means + SD). A two-tailed unpaired *t*-test was applied. *P < 0.05 and **P < 0.01 indicate significant differences compared with the ds*GFP* control group

Among the various types of pigmentation in insects, melanin-type pigment is prevalent [33, 34]. RNA-seq analysis after RNAi targeting *Nllw* revealed a total of 184 differentially expressed genes (DEGs) in 0 h-female adults, comprising 35 down-regulated and 149 up-regulated genes, compared with the ds*GFP* control group (Supplementary Table 1 A, B). Moreover, Gene Ontology (GO) analysis indicated that 3 genes related to the regulation of the circadian sleep/wake cycle (GO:0042749), which was consistent with the classical function of opsin [21], 3 genes associated with positive regulation of morphogenesis of an epithelium (GO:1,905,332), and 2 genes involved in negative regulation of glucose transmembrane transport (GO:0010829) were down-regulated. In comparison, the up-regulated GO terms were primarily linked to various catabolic processes of organic compounds (Fig. 5A, Supplementary Table 2 A, B).

Particularly, a *tyrosine hydroxylase* gene (*NlTH*) and an *arylalkylamine-N-acetyltransferases* gene (*NlaaNAT*) in *N. lugens* were up- and down-regulated after knockdown of *Nllw*, respectively. The *TH* and *aaNAT* were key genes involved in the tyrosine-mediated melanism pathway [35]. Consistent expression levels of *NlTH* and *NlaaNAT* were verified by qRT-PCR with the newly emerging insects after *Nllw* knockdown (Fig. 5B).

## Discussion

As a kind of signal-transducing molecule, opsins play key roles in receiving environmental light signals and subsequently translating them into chemical-free energy [6, 9, 36]. Apart from the typical visual function, *Nllw* is found to be involved in the melanization of body color in *N. lugens* via the tyrosine-mediated melanism pathway, which is important for insect pigmentation [37]. In general, the tyrosine-mediated melanism pathway begins with the hydroxylation of tyrosine into 3,4-dihydroxyphenylalanine (DOPA) under the action of TH [38], followed by decarboxylation into dopamine. Afterward, a series of subsequent oxidation and polymerization reactions result in the formation of melanin from DOPA or dopamine [37]. Different from the TH, the aaNAT acts as a melanin-suppressing factor by *N*-acetylating dopamine to form *N*-acetyldopamine (NADA), a major precursor for colorless sclerotin [35]. To date, both *TH* and *aaNAT* involved in melanization have been extensively studied in several insects, of them, the loss of *TH* function is suggested to diminish black pigmentation [39, 40]; while the loss of *aaNAT* function contributes to dark coloration [41, 42]. In this study, RNAi against *Nllw* up-regulated the expression of *NlTH* and down-regulated that of *NlaaNAT*, which suggested that *Nllw* participated in regulating the changes in melanin precursor substances, resulting in the excessive accumulation of DOPA or dopamine and ultimately the black individuals, as observed in *B. mori* [43]. Interestingly, such result is consistent with the neuropeptide, *Elevenin*, as well as its receptor (*NlA42*) in *N. lugens*: when either *Elevenin* or *NlA42* is knocked down, the tyrosine-mediated cuticle melanism pathway is activated with up-regulation of *NlTH* and down-regulation of *NlaaNAT*, thus inducing melanization, furthermore, the *Elevenin* was scarcely expressed in integument but extremely highly expressed in brain, while its receptor *NlA42* was mainly expressed in both of brain and integument [44]. Similarly, the tissue-expression patterns showed that *Nllw* was extremely highly expressed in eyes but had a low expression level in integuments, consistent with a previous report indicating that *Nllw* is expressed in compound and ocelli eyes of *N. lugens* by in-situ hybridization [14].

A variety of body colors are observed in wild-type *N. lugens*, with predominant colors being black and light which displays yellow or brown. Furthermore, as suggested in a survey, the dominant body color of *N. lugens* in Guangzhou, China, varies across different periods [45, 46]. Additionally, under our phytotron conditions, the majority of *N. lugens* exhibit a brown coloration, with only a minority displaying a black coloration [44]. This observation confirms that body color polyphenism is an important phenomenon, alongside the wing polyphenism in *N. lugens* [28]. The diversity of body colors enables insects to better adapt to different environments. For instance, the black body color achieves the advantageous purpose of ecological adaptation to climatic conditions under low temperatures [47]. In the meantime, melanization takes part in the innate immune system to resist pathogens and non-self-substances, that are involved in a phenoloxidase [48]. However, previous studies have indicated that distinct photoperiod does not significantly affect the changes in body color composition of *N. lugens*, and less than half of the black 5th -instar nymphs retain their black coloration after molting [44]. Typically, the phenomenon of melanization in *Biston betularia* (Lepidoptera) is a well-known example that highlights the crucial role of visual perception in body color change [49]. While in the case of *N. lugens*, the regulatory mechanism underlying body color may be much more intricate than our current understanding, just like in *Locusta migratoria* (Orthoptera), the pigment of body color is complex and can be modified by various factors, such as endogenous hormone and population density [50]. Moreover, previous research has demonstrated that both body color and wing morphology of *N. lugens* contribute to the regulation of development [51]. *N. lugens* is widely distributed among rice-growing regions in the tropical and temperate Asian Pacific region [52], and there are variations in migration and reproductive preferences among different regional populations of *N. lugens* [53, 54]. Consequently, it is important to conduct extensive investigations into the relationship between body color and the environment in various regions, to provide further insights into the polyphenism of body color in *N. lugens*. In a word, it is speculated that *Nllw* is not only responsible for long-wave spectrum absorption but also plays a crucial role in the complex regulatory network between external signals and body color via the tyrosine signal pathway, with the regulation of key enzymes *NlTH* and *NlaaNAT*.

In addition, a predicted *NlUV3-like* was identified in *N. lugens*, but the *B-sensitive opsin* was lacking, which was different from *Nephotettix cincticeps*, another rice pest in Hemiptera, that possesses classic trichromacy [14]. Notably, the down-regulation of *Nllw* led to the up-regulation of *NlUV1*/*2*, and vice versa, suggesting partial functional compensation between *Nllw* and *NlUV1*/*2*. However, the expression of *NlUV3-like* was independent of the other three opsins, aligning with the phylogenetic analysis result. Additionally, as revealed by the phylogenetic tree analysis, NlUV1/2 exhibited higher conservation within the Delphacidae family, whereas NlUV3-like diverged from NlUV1/2. Until now, the mechanisms of vision in *N. lugens* remain unknown, including whether the replicated UV-sensitive opsins can compensate for the missing B-sensitive opsin, like that in beetles [18]. Besides, although the silencing of any one of the *Nllw*, *NlUV1/2*, and *NlUV3-like* did not lead to significant mortality, it did not necessarily imply that it has no impact on the fitness of *N. lugens*. As crucial components of visual pigments, the changes in preference for spectral sensitivity in *N. lugens* after the silencing of the four opsins are still unknown. Given that the *N. lugens* is a kind of long-distance migratory insect with a specialized feeding on rice, the influence of the spectral preference deserves further investigation. For example, a study in monarch has demonstrated that the specific opsin expansions are involved in sensing skylight cues [55]. Additionally, there is also another opsin reported in *Ciona intestinalis* (Phlebobranchia), which plays an important role in photoresponse during swimming behavior [56].

## Conclusions

In summary, this study has highlighted an unconventional role of one opsin (Nllw) in the regulation of cuticle melanization through tyrosine signal pathway, implying a cross-talk between the gene pathways underlying the visual system and the morphological differentiation in insects and a complex mechanism existed in them. Besides, another UV-like opsin (NlUV3-like), which was homolog to the Rh7 in *Drosophila* was described, and the detailed study of *Nllw*, *NlUV1/2*, and *NlUV3-like* revealed the conservation and evolution of opsins in *N. lugens*.

## Methods

### Insect rearing

*N. lugens* used in this study is reared on the Xiushui rice plant in a phytotron under 26.5℃, 65–75% relative humidity, and a 14/10 h light/dark photoperiod at Ningbo University, Ningbo, China.

### Sequence analysis

DNAMAN software was used to predict gene CDS. NCBI blast (https://blast.ncbi.nlm.nih.gov/Blast.cgi) and NCBI CD search (https://www.ncbi.nlm.nih.gov/Structure/cdd/wrpsb.cgi) websites were used to reveal potential gene function and conserved domains. Protein sequences were aligned using Clustal X software. Furthermore, through the NCBI Splign website (https://www.ncbi.nlm.nih.gov/sutils/splign/splign.cgi?textpage=online&level=form), the distribution of gene CDS on *N. lugens* genomic sequence was displayed.

### Phylogenetic analysis

28 amino acid sequences from ten insect species of five orders were downloaded from the NCBI database (Supplementary Table 3). A phylogenetic tree was thereby constructed based on Nllw, NlUV1/2, NlUV3-like, and the above 28 proteins with the software MEGA7, following the maximum likelihood method with 1000 bootstrap replications [57]. Furthermore, the NlaaNAT amino acid sequence was selected as an outgroup member.

### Real-time quantitative PCR (qRT-PCR) analysis

The total RNA of the target sample was extracted using the Trizol RNA isolater Total RNA Extraction Reagent (Vazyme Biotech, Nanjing, China) following the manufacturer’s protocol. The quality and concentration of the isolated RNAs were assessed using a NanoDrop One Spectrometer (Thermo Fisher Scientific, USA). Next, 400 ng of each RNA was reverse transcripted into cDNA using the HiScript II Q RT SuperMix (+ gDNA wiper) kit (Vazyme Biotech, Nanjing, China) in line with the protocol. Finally, qRT-PCR was carried out with an ABI QuantStudio 5 equipment (Thermo Fisher Scientific, USA) in a 10-µL reaction system, containing 5µL of ChamQ SYBR Color qPCR Master Mix (Vazyme Biotech, Nanjing, China), 2.4µL of ddH_2_O, 2µL of 20-fold diluted cDNA, and 0.3µL of each 10 µmol primer. The procedure of qPCR comprised an initial denaturation step at 95 °C for 5 min, followed by 40 cycles of denaturation at 95 °C for 10 s, and primer annealing together with amplification at 60 °C for 30 s. With 2^−ΔΔCt^ (Ct: cycle threshold) method [58], the relative expression level of the target gene was calculated, with *N. lugens 18 S ribosomal RNA* (*Nl18S*, GenBank accession number: JN662398.1) serving as the housekeeping gene. Primers used for qRT-PCR are listed in (Supplementary Table 4 A).

### Spatiotemporal expression analysis

Temporal expression patterns of *Nllw*, *NlUV1/2*, and *NlUV3-like* were analyzed with the FPKM values. Data from three biological replicates of each sample were used (Supplementary Table 5 A). By dissecting different tissues containing integument, digestive tract, ovary, testis, fat body, and eye from the 5th instar nymphs and adults, the *Nllw*, *NlUV1/2*, and *NlUV3-like* expression patterns in tissues were analyzed by qRT-PCR. Data from three biological replicates are listed in (Supplementary Table 5B).

### RNA interference (RNAi)

One specific sequence of the target gene was cloned to synthesize the double-stranded RNA (dsRNA) with a MEGAscript T7 Transcription Kit (Ambion, Austin, TX), following the manufacturer’s protocol. The *green fluorescent protein* (*GFP*) gene from *Aequorea victoria* (Leptothecata) was used as a negative control. Primers containing the T7 promoter sequences used for cloning are listed in (Supplementary Table 4B). With a FemtoJet Microinjection system (Eppendorf, North America), around 250 ng of dsRNA was injected into each of the 4th instar nymphs. The gene silencing efficiency was detected at 72 h post-injection by randomly selecting five insects from each sample. The insect survival rate after RNAi was counted for 10 days and the insect phenotypes were recorded using a stereomicroscope (Leica 151 S8AP0, Germany).

### Transcriptome sequencing (RNA-seq)

Firstly, dsRNA of *Nllw* was injected into the 3rd instar *N. lugens* nymphs, and female adult *N. lugens* was collected at 0 h after emergence since the body color difference was more pronounced than male adults after RNAi. Afterward, ten individuals were homogenized for total RNA extraction, and ds*GFP*-treated sample was taken as the negative control. Each treatment was replicated thrice for cDNA library preparation and sequencing on the Illumina Novaseq 6000 platform (Novogene, Tianjin, China). Clean reads were aligned to the *N. lugens* reference genome using HISAT2. Furthermore, transcripts per million (TPM) expression values were calculated based on feature counts for genes by Cufflink. DEGs were identified using the DESeq2 package upon the threshold of P-value < 0.05 and log2 ratio > 1. To explore the biological progress of DEGs, GO analysis was conducted with eggNOG, and GO terms were classified as biological process (BP), cellular component (CC), and molecular function (MF) categories.

### Statistical analysis

The data are presented as means + SD in graphic instructions, and three biological repetitions were performed for each experiment. Statistical analysis was performed using GraphPad Prism 7.00 software and Mircosoft Excel. The two-tailed Student’s *t*-test was conducted to compare the difference. The significance level was set at * p < 0.05, ** p < 0.01, and *** p < 0.001.

## Electronic supplementary material

Below is the link to the electronic supplementary material.

Supplementary Material 1

## Data Availability

The transcriptome raw sequence data are available in the Genome Sequence Archive in National Genomics Data Center, China National Center for Bioinformation / Beijing Institute of Genomics, Chinese Academy of Sciences (https://ngdc.cncb.ac.cn/gsub/), with the GSA CRA010945 under Bioproject PRJCA016793.

## References

[1] Tang S, Guo A (2001). Choice behavior of *Drosophila* facing contradictory visual cues. Science.

[2] Giurfa M, Menzel R (1997). Insect visual perception: complex abilities of simple nervous systems. Curr Opin Neurobiol.

[3] Cutler D, Bennett R, Stevenson R, White R (1995). Feeding behavior in the nocturnal moth *Manduca sexta* is mediated mainly by blue receptors, but where are they located in the retina?. J Exp Biol.

[4] Choi N, Adams M, Fowler-Finn K, Knowlton E, Rosenthal M, Rundus A, Santer RD, Wilgers D, Hebets EA (2022). Increased signal complexity is associated with increased mating success. Biol Lett.

[5] Liénard MA, Valencia-Montoya WA, Pierce NE (2022). Molecular advances to study the function, evolution and spectral tuning of arthropod visual opsins. Philos Trans R Soc Lond B Biol Sci.

[6] Shichida Y, Imai H (1998). Visual pigment: G-protein-coupled receptor for light signals. Cell Mol Life Sci.

[7] Nagata T, Koyanagi M, Lucas R, Terakita A (2018). An all-trans-retinal-binding opsin peropsin as a potential dark-active and light-inactivated G protein-coupled receptor. Sci Rep.

[8] Bownds D. Site of attachment of retinal in rhodopsin. Nat 1967, 216(5121):1178–81. 10.1038/2161178a0 4294735

[9] Briscoe AD, Chittka L (2001). The evolution of color vision in insects. Annu Rev Entomol.

[10] Zuker CS, Cowman AF, Rubin GM (1985). Isolation and structure of a rhodopsin gene from *D. melanogaster*. Cell.

[11] Jean-Guillaume CB, Kumar JP (2022). Development of the ocellar visual system in *Drosophila melanogaster*. FEBS J.

[12] Menzel R, Backhaus W (1989). Color vision honey bees: Phenomena and physiological mechanisms. Facets of vision.

[13] Liu Y-J, Yan S, Shen Z-J, Li Z, Zhang X-F, Liu X-M, Zhang Q-W, Liu X-X (2018). The expression of three opsin genes and phototactic behavior of *Spodoptera exigu*a (Lepidoptera: Noctuidae): evidence for visual function of opsin in phototaxis. Insect Biochem Mol Biol.

[14] Matsumoto Y, Wakakuwa M, Yukuhiro F, Arikawa K, Hiroaki N (2014). Attraction to different Wavelength Light Emitting Diodes (LEDs), the compound Eye structure, and opsin genes in *Nilaparvata lugens*. JPN J APPL ENTOMOL Z.

[15] Van Der Kooi CJ, Stavenga DG, Arikawa K, Belušič G, Kelber A (2021). Evolution of insect color vision: from spectral sensitivity to visual ecology. Annu Rev Entomol.

[16] Futahashi R, Kawahara-Miki R, Kinoshita M, Yoshitake K, Yajima S, Arikawa K. Fukatsu TJPotNAoS: extraordinary diversity of visual opsin genes in dragonflies. 2015, 112(11):E1247–56. 10.1073/pnas.1424670112 PMC4371951 25713365

[17] Jackowska M, Bao R, Liu Z, McDonald EC, Cook TA, Friedrich M (2007). Genomic and gene regulatory signatures of cryptozoic adaptation: loss of blue sensitive photoreceptors through expansion of long wavelength-opsin expression in the red flour beetle *Tribolium castaneum*. Front Zool.

[18] Sharkey CR, Fujimoto MS, Lord NP, Shin S, McKenna DD, Suvorov A, Martin GJ, Bybee SM (2017). Overcoming the loss of blue sensitivity through opsin duplication in the largest animal group, beetles. Sci Rep.

[19] Papatsenko D, Sheng G, Desplan C (1997). A new rhodopsin in R8 photoreceptors of *Drosophila*: evidence for coordinate expression with Rh3 in R7 cells. Development.

[20] Huber A, Schulz S, Bentrop J, Groell C, Wolfrum U, Paulsen R (1997). Molecular cloning of *Drosophila* Rh6 rhodopsin: the visual pigment of a subset of R8 photoreceptor cells. FEBS Lett.

[21] Ni JD, Baik LS, Holmes TC, Montell C. A rhodopsin in the brain functions in circadian photoentrainment in *Drosophila*. Nat 2017, 545(7654):340–4. 10.1038/nature22325 PMC5476302 28489826

[22] Frentiu FD, Yuan F, Savage WK, Bernard GD, Mullen SP, Briscoe AD (2015). Opsin clines in butterflies suggest novel roles for insect photopigments. Mol Biol Evol.

[23] McCulloch KJ, Macias-Muñoz A, Briscoe AD (2022). Insect opsins and evo-devo: what have we learned in 25 years?. Philos Trans R Soc Lond B Biol Sci.

[24] Shen WL, Kwon Y, Adegbola AA, Luo J, Chess A, Montell C (2011). Function of rhodopsin in temperature discrimination in *Drosophila*. Science.

[25] Sokabe T, Chen H-C, Luo J, Montell C (2016). A switch in thermal preference in *Drosophila* larvae depends on multiple rhodopsins. Cell Rep.

[26] Li Q, DeBeaubien NA, Sokabe T, Montell C (2020). Temperature and sweet taste integration in *Drosophila*. Curr Biol.

[27] Briscoe AD, Bybee SM, Bernard GD, Yuan F, Sison-Mangus MP, Reed RD, Warren AD, Llorente-Bousquets J, Chiao C-C (2010). Positive selection of a duplicated UV-sensitive visual pigment coincides with wing pigment evolution in *Heliconius butterflies*. Proc Natl Acad Sci U S A.

[28] Xu H-J, Xue J, Lu B, Zhang X-C, Zhuo J-C, He S-F, Ma X-F, Jiang Y-Q, Fan H-W, Xu J-Y (2015). Two insulin receptors determine alternative wing morphs in planthoppers. Nature.

[29] Xue J, Bao Y-Y, Li B-l, Cheng Y-B, Peng Z-Y, Liu H, Xu H-J, Zhu Z-R, Lou Y-G, Cheng J-A (2010). Transcriptome analysis of the brown planthopper *Nilaparvata lugens*. PLoS ONE.

[30] Ye YX, Zhang HH, Li DT, Zhuo JC, Shen Y, Hu QL, Zhang CX (2021). Chromosome-level assembly of the brown planthopper genome with a characterized Y chromosome. Mol Ecol Resour.

[31] Gärtner W (2000). Invertebrate visual pigments. Molecular Mechanisms in Visual Transduction.

[32] Lu JB, Guo JS, Chen X, Cheng C, Luo XM, Zhang XY, Moussian B, Chen JP, Li JM, Zhang CX (2022). Chitin synthase 1 and five cuticle protein genes are involved in serosal cuticle formation during early embryogenesis to enhance eggshells in *Nilaparvata lugens*. Insect Sci.

[33] Manickam S. Complexities of cuticular pigmentation in insects. Pigment Cell Melanoma Res 2009, 22(5):523–5. 10.1111/j.1755-148X.2009.00608.x 19614888

[34] Popadić A, Tsitlakidou D (2021). Regional patterning and regulation of melanin pigmentation in insects. Curr Opin Genet Dev.

[35] Liu J, Lemonds TR, Marden JH, Popadić A (2016). A pathway analysis of melanin patterning in a hemimetabolous insect. Genetics.

[36] Zuker CS, Montell C, Jones K, Laverty T, Rubin GM (1987). A rhodopsin gene expressed in photoreceptor cell R7 of the Drosophila eye: homologies with other signal-transducing molecules. J Neurosci.

[37] Arakane Y, Noh MY, Asano T, Kramer KJ. Tyrosine Metabolism for Insect Cuticle Pigmentation and Sclerotization. In: Extracellular Composite Matrices in Arthropods. Edited by Cohen E, Moussian B. Cham: Springer International Publishing Switzerland; 2016: 165–220.

[38] Gorman MJ, Arakane Y (2010). Tyrosine hydroxylase is required for cuticle sclerotization and pigmentation in *Tribolium castaneum*. Insect Biochem Mol Biol.

[39] Liu J, Lemonds TR, Popadić A (2014). The genetic control of aposematic black pigmentation in hemimetabolous insects: insights from *Oncopeltus fasciatus*. Evol Dev.

[40] Ninomiya Y, Tanaka K, Hayakawa Y (2006). Mechanisms of black and white stripe pattern formation in the cuticles of insect larvae. J Insect Physiol.

[41] Qiao L, Li Y, Xiong G, Liu X, He S, Tong X, Wu S, Hu H, Wang R, Hu H (2012). Effects of altered catecholamine metabolism on pigmentation and physical properties of sclerotized regions in the silkworm melanism mutant. PLoS ONE.

[42] Zhan S, Guo Q, Li M, Li M, Li J, Miao X, Huang Y (2010). Disruption of an N-acetyltransferase gene in the silkworm reveals a novel role in pigmentation. Development.

[43] Dai F-Y, Qiao L, Tong X-L, Cao C, Chen P, Chen J, Lu C, Xiang Z-H (2010). Mutations of an arylalkylamine-N-acetyltransferase, Bm-iAANAT, are responsible for silkworm melanism mutant. J Biol Chem.

[44] Wang SL, Wang WW, Ma Q, Shen ZF, Zhang MQ, Zhou NM, Zhang CX (2019). Elevenin signaling modulates body color through the tyrosine-mediated cuticle melanism pathway. FASEB J.

[45] Chen H-Q. Study on the body color differentiation of brown planthopper. Sun Yat-Sen University; 2005.

[46] Galolo ARV, Torres MAJ, Demayo CG. Paramere morphology of two colorrmorphs of the Brown Planthopper *Nilaparvata lugens* (Stål)(Homoptera: Delphacidae). pp. In: 2011 *2nd International Conference on Environmental Science and Technology; Singapore*. IACSIT Press 2011: 370.

[47] Lo Pinto M, Guarino S, Agrò A (2023). Evidence of Seasonal Variation in body color in adults of the Parasitoid *Cirrospilus pictus* (Hymenoptera: Eulophidae) in Sicily, Italy. Insects.

[48] Marieshwari BN, Bhuvaragavan S, Sruthi K, Mullainadhan P, Janarthanan S (2022). Insect phenoloxidase and its diverse roles: melanogenesis and beyond. J Comp Physiol B.

[49] Noor MA, Parnell RS, Grant BS (2008). A reversible color polyphenism in american peppered moth (*Biston betularia cognataria*) caterpillars. PLoS ONE.

[50] Tanaka S (2000). Hormonal control of body-color polymorphism in *Locusta migratoria*: interaction between [His7]-corazonin and juvenile hormone. J Insect Physiol.

[51] Morooka S, Tanaka Y, Tojo S (2011). Genetic variation in developmental times among four pure lines exhibiting specific wing form and body color in the brown planthopper, *Nilaparvata lugens* (Hemiptera: Auchenorrhyncha). Appl Entomol Zool.

[52] Hu Q-l, Zhuo J-C, Ye Y-X, Li D-T, Lou Y-H, Zhang X-Y, Chen, Wang S-L, Wang Z-C, Lu J-B, et al. Whole genome sequencing of 358 brown planthoppers uncovers the landscape of their migration and dispersal worldwide. bioRxiv preprint. 2019. https://doiorg/101101/798876.

[53] Wada T, Ito K, Takahashi A, Tang J (2009). Starvation tolerance of macropter brown planthopper, *Nilaparvata lugens*, from temperate, subtropical, and tropical populations in East and South-East Asia. Entomol Exp Appl.

[54] Wada T, Policy. Rice Planthoppers in Tropics and Temperate East Asia: difference in their Biology. In: Rice Planthoppers: Ecology, Management, Socio Econ 2015: 77–89.

[55] Zhan S, Merlin C, Boore JL, Reppert SM (2011). The monarch butterfly genome yields insights into long-distance migration. Cell.

[56] Inada K, Horie T, Kusakabe T, Tsuda M (2003). Targeted knockdown of an opsin gene inhibits the swimming behaviour photoresponse of ascidian larvae. Neurosci Lett.

[57] Kumar S, Stecher G, Tamura K (2016). MEGA7: molecular evolutionary genetics analysis version 7.0 for bigger datasets. Mol Biol Evol.

[58] Livak KJ, Schmittgen TD (2001). Analysis of relative gene expression data using real-time quantitative PCR and the 2^– ∆∆CT^ method. Methods.

